# Supplementary material for: Regulation of the cell division hydrolase RipC by the FtsEX system in Mycobacterium tuberculosis
Source: Nat Commun. 2023 Dec 4;14:7999. doi: 10.1038/s41467-023-43770-6 (PMC10694151; doi:10.1038/s41467-023-43770-6)
Supplement: Supplementary file 1 — Supplementary information [file 41467_2023_43770_MOESM1_ESM.pdf]

Supplementary information for

Regulation of the cell division hydrolase RipC by the FtsEX system in *Mycobacterium tuberculosis*

Jianwei Li[1, #], Xin Xu[1, #], Jian Shi[2], Juan A. Hermoso[3, *], Lok-To Sham[4, *], and Min Luo[1, 2, *]

[1] Department of Biological Sciences, Faculty of Science, National University of Singapore
[2] Center for Bioimaging Sciences, Department of Biological Sciences, National University of Singapore
[3] Department of Crystallography and Structural Biology, Instituto de Química-Física "Blas Cabrera", Consejo Superior de Investigaciones Científicas, Madrid, Spain
[4] Infectious Diseases Translational Research Programme and Department of Microbiology and Immunology, Yong Loo Lin School of Medicine, National University of Singapore
[#] These authors contributed equally: Jianwei Li, Xin Xu
*Corresponding author. **Email:** xjuan@iqfr.csic.es, lsham@nus.edu.sg and dbslmin@nus.edu.sg

This PDF file includes:

Supplementary Figures 1 to 12
Supplementary Tables 1 to 2

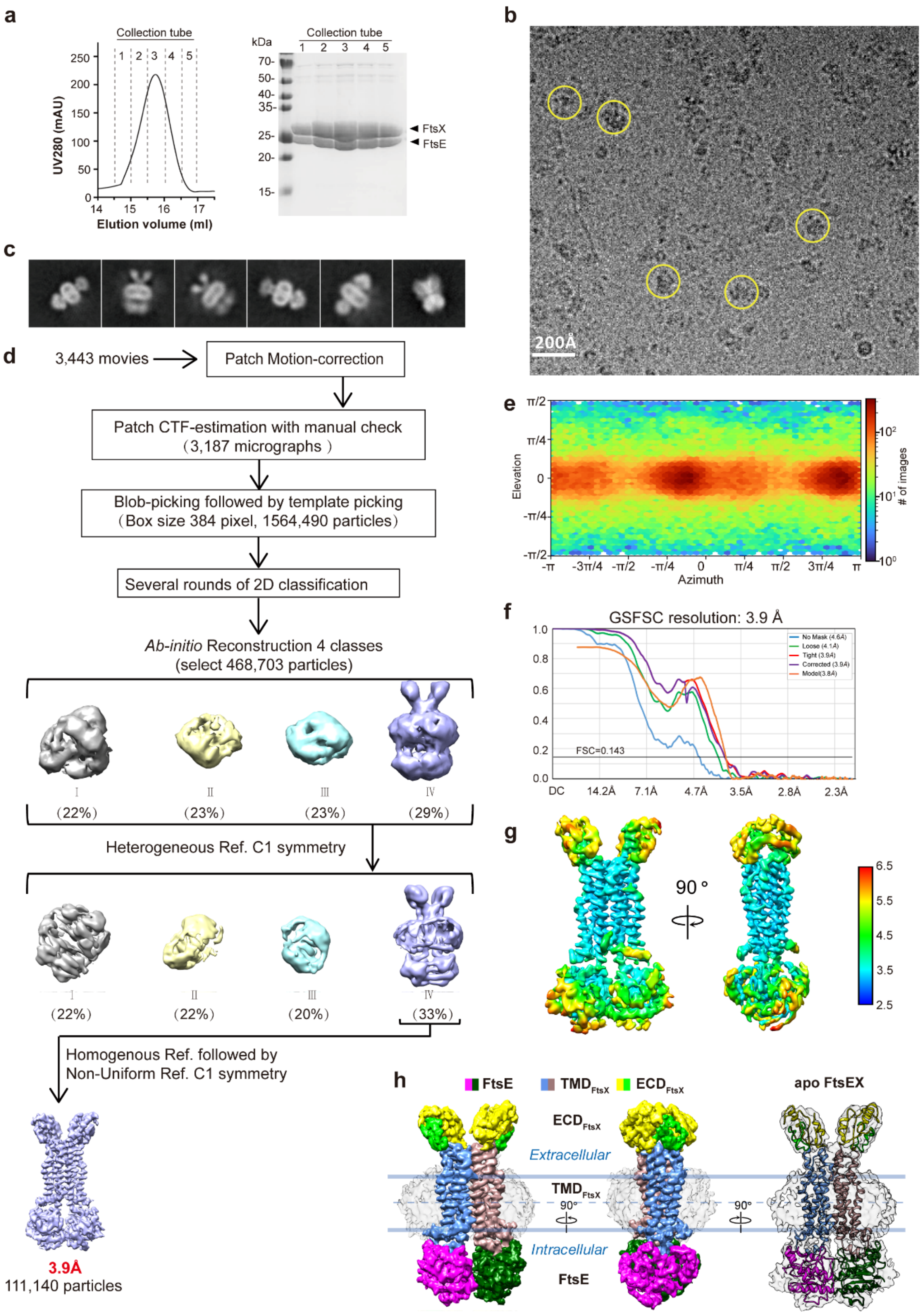

Supplementary Figure 1. Biochemical reconstitution and single-particle cryo-EM analysis of FtsEX complex. a, SEC profile and SDS-PAGE gel of purified FtsEX reconstituted in peptidiscs.

Source data are provided as a Source Data file. **b,** Representative cryo-EM image with several particles marked by yellow circles. **c,** Representative 2D averages of cryo-EM particle images. The box dimension is 340 Å. **d,** Image processing flowchart. The final maps of one major conformation with its overall resolutions is indicated in red. **e,** Angular distribution of the cryo-EM particles included in the final 3D reconstruction. **f,** The Fourier shell correlation (FSC) curve: gold standard FSC between two half data maps or model with the final map, with indicated resolution at FSC=0.143 (FSC corrected applied). Source data are provided as a Source Data file. **g,** The cryo-EM map filtered to the estimated overall resolution and colored according to local resolution. **h** Front- and side-views of the cryo-EM density map of FtsEX in the absence of ATP (left and middle), and with the built model shown (right). Color scheme: TMD_{FtsX} in cornflower blue and rosy brown, the upper lobe of ECD_{FtsX} in yellow, and the lower lobe of ECD_{FtsX} in green, FtsE in magenta and dark green.

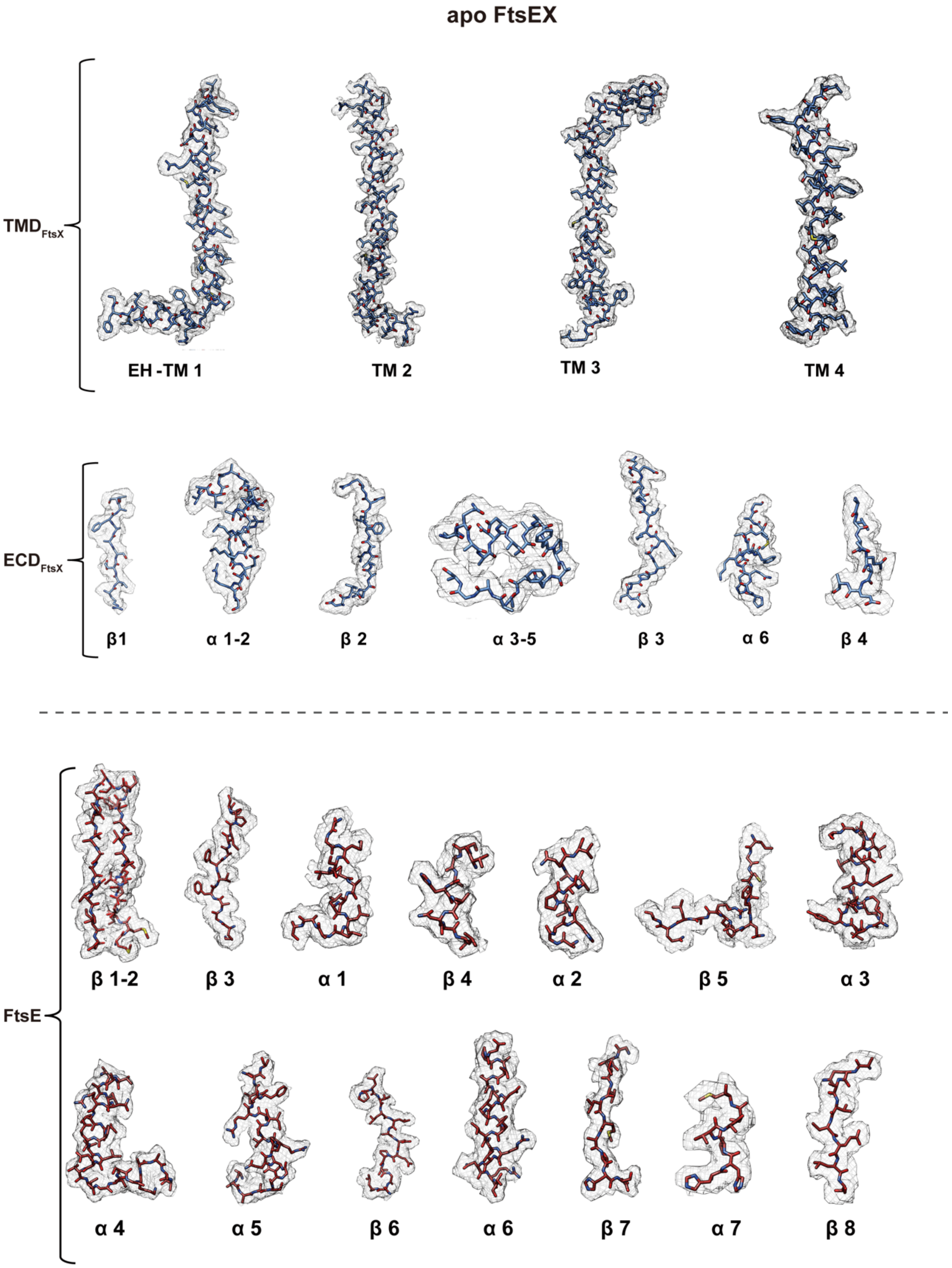

Supplementary Figure 2. Cryo-EM density of different regions of FtsE and FtsX in the structure of FtsEX complex.

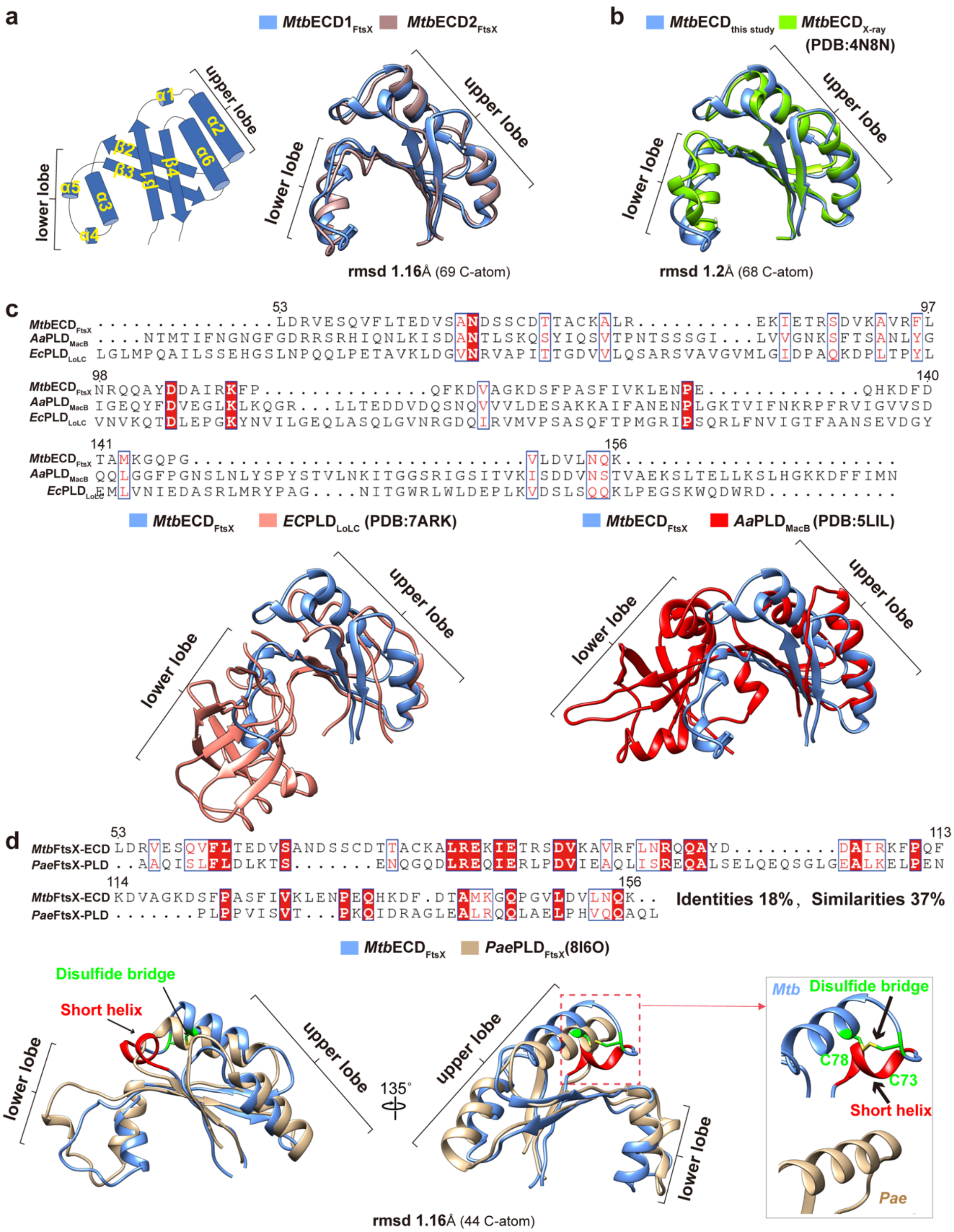

Supplementary Figure 3. Structural comparison of ECD monomers. a, Left: Topology diagram of ECD domain in *Mtb*FtsEX. α helices are shown as cylinder, β sheets are shown as arrows; Right: Structural comparison between the two ECD domains in *Mtb*FtsEX, with the structures overlaid on each other. **b,** Comparison of the ECD monomer structure with a previously determined X-ray structure (PDB code: 4N8N [https://www.rcsb.org/structure/4N8N]). **c,** Sequence alignment and structural comparison of the ECD/PLD from *Mtb*FtsX, *Ec*LolC (PDB code: 7ARK

[https://www.rcsb.org/structure/7ARK]), and *Aa*MacB (PDB code: 5LIL [https://www.rcsb.org/structure/5LIL]). **d,** Sequence alignment and structural comparison of the ECD/PLD from *Pae*FtsX (PDB code: 8I6O [https://www.rcsb.org/structure/8I6O]) and *Mtb*FtsX. In *Mtb*FtsEX, the extra short helix and the disulfide bridge are labeled in red and green, respectively.

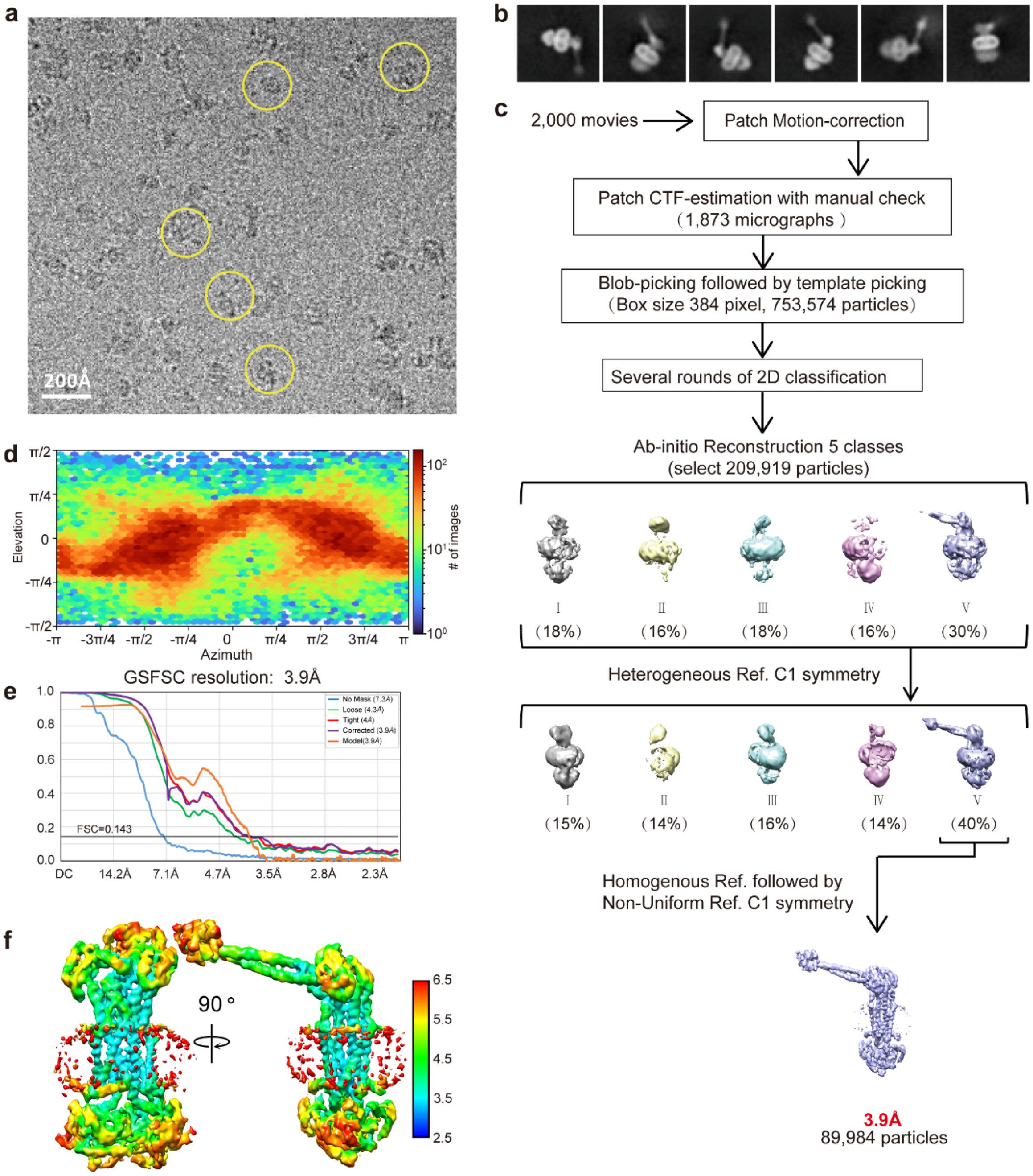

Supplementary Figure 4. Single-particle cryo-EM analysis of FtsEX-RipC complex in the absence of ATP. a, Representative cryo-EM image with several particles marked by circles. **b,** 2D averages of cryo-EM particle images. The box dimension is 400 Å. **c,** Image processing flowchart. The final maps of one major conformation with its overall resolution is indicated in red. **d,** Angular distribution of the cryo-EM particles included in the final 3D reconstruction. **e,** The Fourier shell correlation (FSC) curve: gold standard FSC between two half data maps or the final model with the EM map, with indicated resolution at FSC=0.143 (FSC corrected applied). Source data are provided as a Source Data file. **f,** The surface cryo-EM map was filtered to the estimated overall resolution and colored according to local resolution.

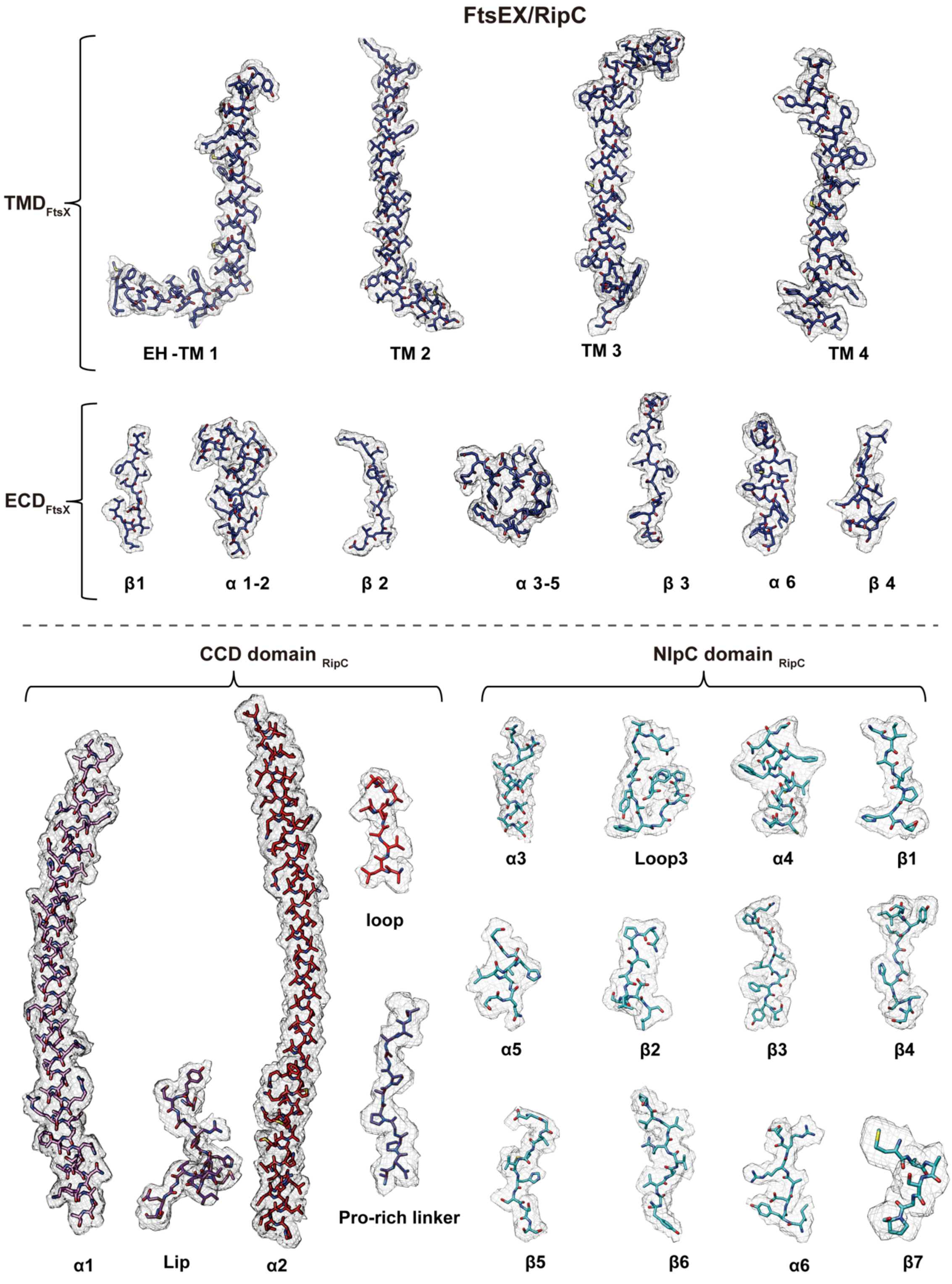

Supplementary Figure 5. Cryo-EM density of different regions of FtsX and RipC in the structure of FtsEX-RipC complex.

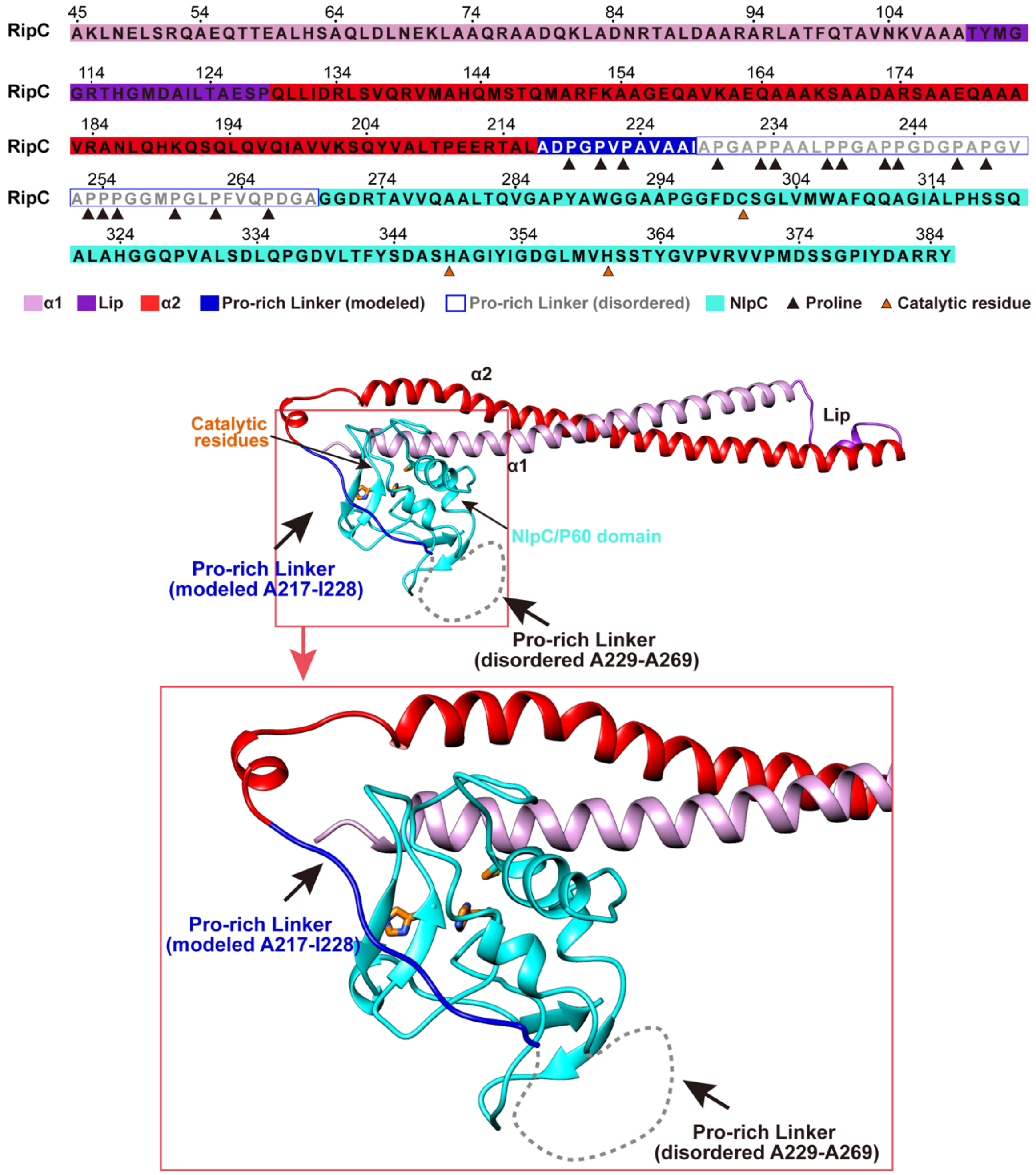

Supplementary Figure 6. Sequence and structure analysis of RipC in an autoinhibited state. The structure is color-coded to highlight different domains, including the α1 domain in plum, lip in purple, α2 in red, and the NlpC/P60 catalytic domain in cyan. The modeled Pro-rich linker is shown in blue, while the disordered Pro-rich linker is denoted by a dashed line. The catalytic residues are highlighted in orange. The Pro-rich linker contains 18 proline residues, as denoted by black triangles underneath.

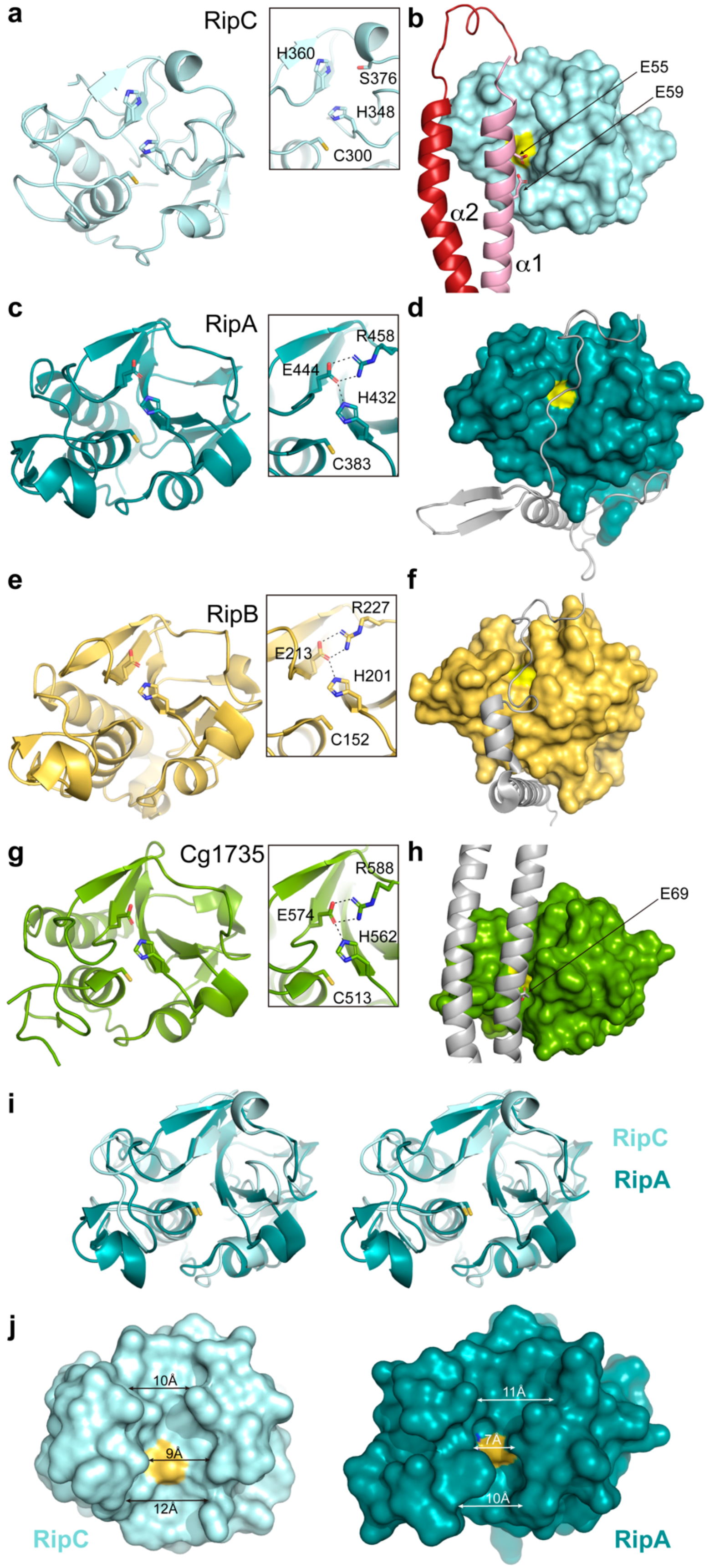
a
RipC
H360
S376
H348
C300
b
E55
E59
α2
α1
c
RipA
R458
E444
H432
C383
d
e
RipB
R227
E213
H201
C152
f
g
Cg1735
R588
E574
H562
C513
h
E69
i
RipC
RipA
j
10Å
9Å
12Å
RipC
11Å
7Å
10Å
RipA

Supplementary Figure 7. Comparison of RipC catalytic domain with structural homologs. **a,** Cartoon representation of the catalytic domain of RipC (this work). The zoomed area shows the catalytic triad Cys-His-His (C300, H348, H360) observed in the NlpC/P60 family; this triad is not conserved in RipA homologues. **b,** α1 helix in RipC blocks the active site of RipC in an auto-inhibited conformation with two glutamate residues (E55, E59) interacting with residues of the catalytic domain. **c,** Cartoon representation of the catalytic domain of RipA (PDB code: 3NE0 [https://www.rcsb.org/structure/3NE0]). The zoomed area shows catalytic residues represented as capped sticks. **d,** The N-terminal segment of RipA (residues 260-321) blocks the active site of RipA. **e,** Cartoon representation of the catalytic domain of RipB (PDB code: 3PBI [https://www.rcsb.org/structure/3PBI]). The zoomed area shows catalytic residues represented as capped sticks. **f,** The N-terminal segment of RipB (residues 30-97) blocks the active site of RipB. **g,** Cartoon representation of the catalytic domain of Cg1735 (PDB code: 8AUC [https://www.rcsb.org/structure/8AUC]). The zoomed area shows catalytic residues represented as capped sticks. **h,** Two helices from the coiled-coil domain of a symmetry-related molecule block the active site of Cg1735; residue E69 from the coiled-coil helix makes an H-bond with the catalytic C513. **i,** Stereo view showing the changes in the loops around the catalytic center of RipC and RipA (catalytic Cys residue represented as capped sticks). **j,** Molecular surface representation of RipC and RipA. Position of the catalytic Cys residue is highlighted in yellow. Active sites of both enzymes present relevant differences in shape and dimensions.

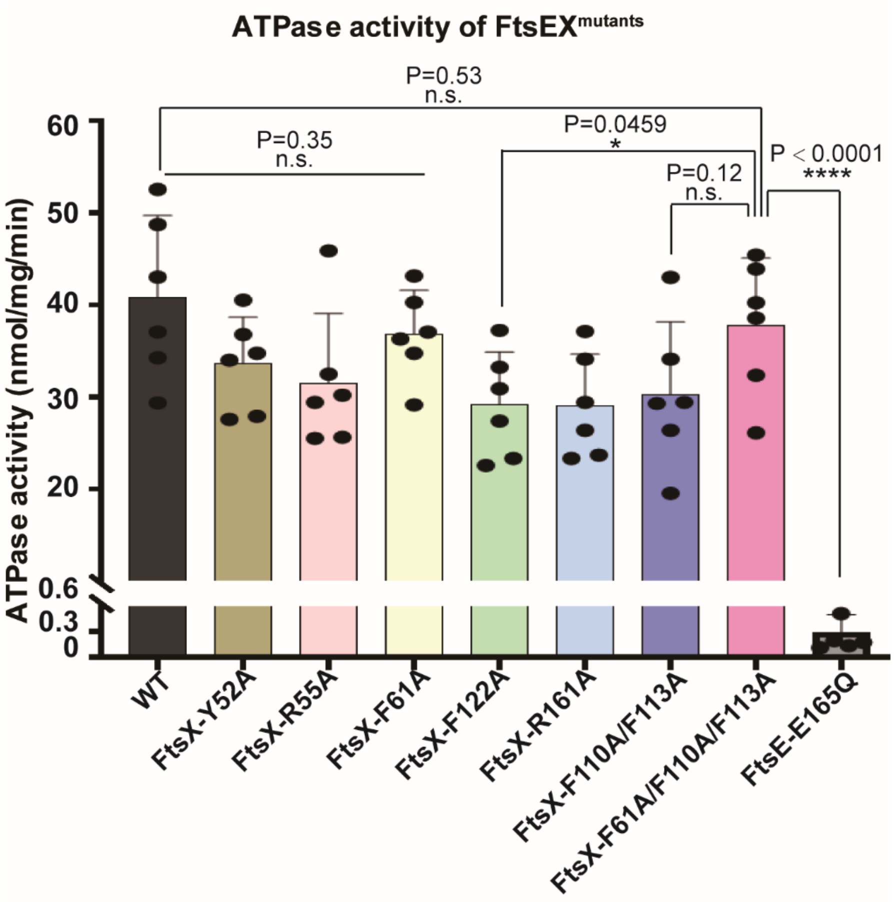

Supplementary Figure 8. ATPase activity of FtsEX mutants. Inhibition of the ATPase activity of FtsEX was observed to a minor extent upon mutation of residues involved in the interaction between FtsX and RipC. The mutant FtsE^{E165Q}X serves as a negative control. n=6 replicates, error bars were presented as mean ± SD. Two-tailed unpaired t test was applied; *P<0.05, ****P < 0.0001, n.s. no significant difference. The exact P values are shown in figure and source data are provided as a Source Data file.

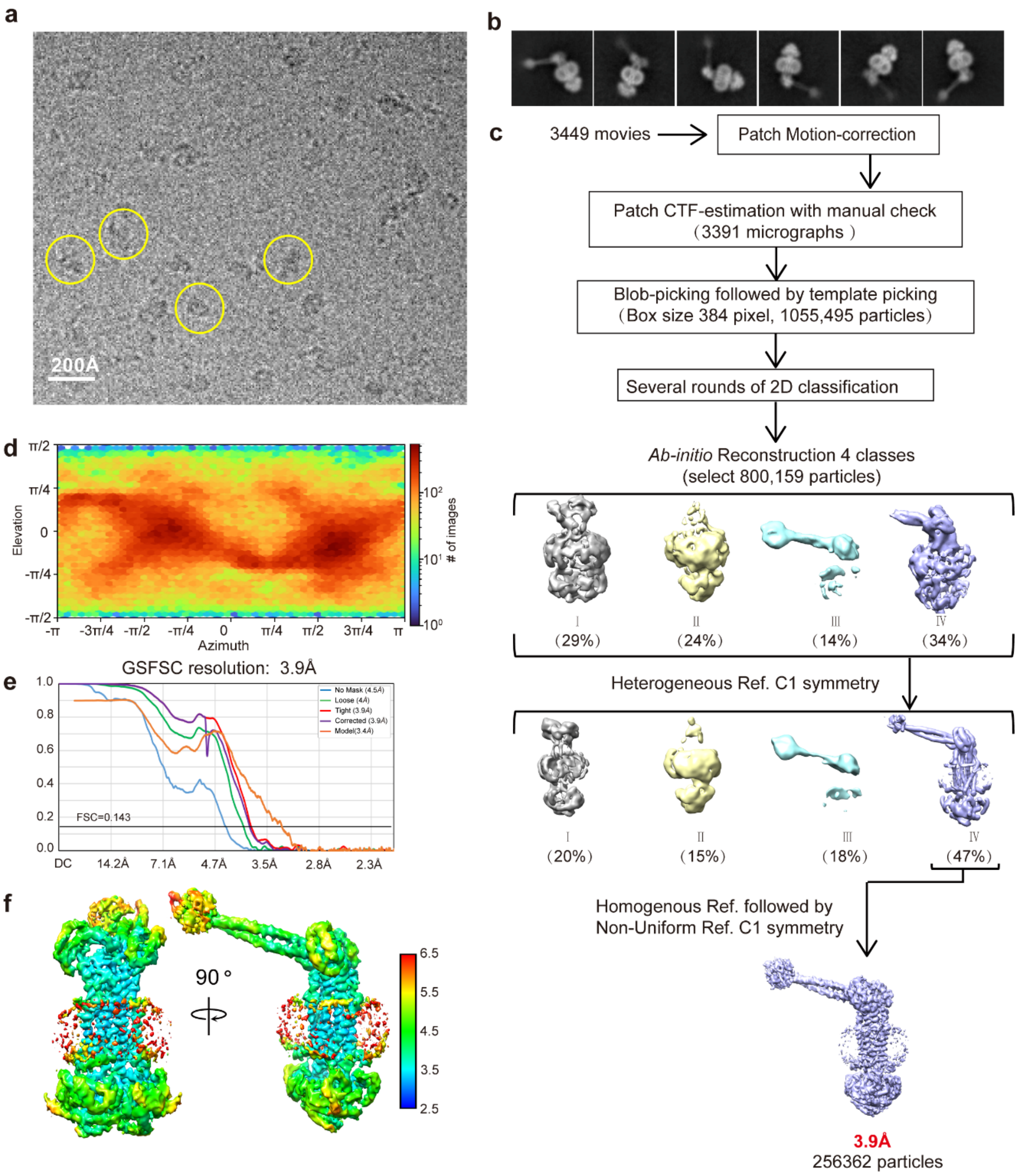

Supplementary Figure 9. Single-particle cryo-EM analysis of FtsE^{E165Q}X-RipC complex in the presence of ATP. a, Representative cryo-EM image with several particles marked by yellow circles. **b,** 2D averages of cryo-EM particle images. The box dimension is 400 Å. **c,** Image processing flowchart. The final maps of one major conformation with its overall resolution are indicated in red. **d,** Angular distribution of the cryo-EM particles included in the final 3D reconstruction. **e,** The Fourier shell correlation (FSC) curve: gold standard FSC between two half data maps with indicated resolution at FSC=0.143 (FSC corrected applied). Source data are provided as a Source Data file. **f,** The surface cryo-EM map was filtered to the estimated overall resolution and coloured according to local resolution.

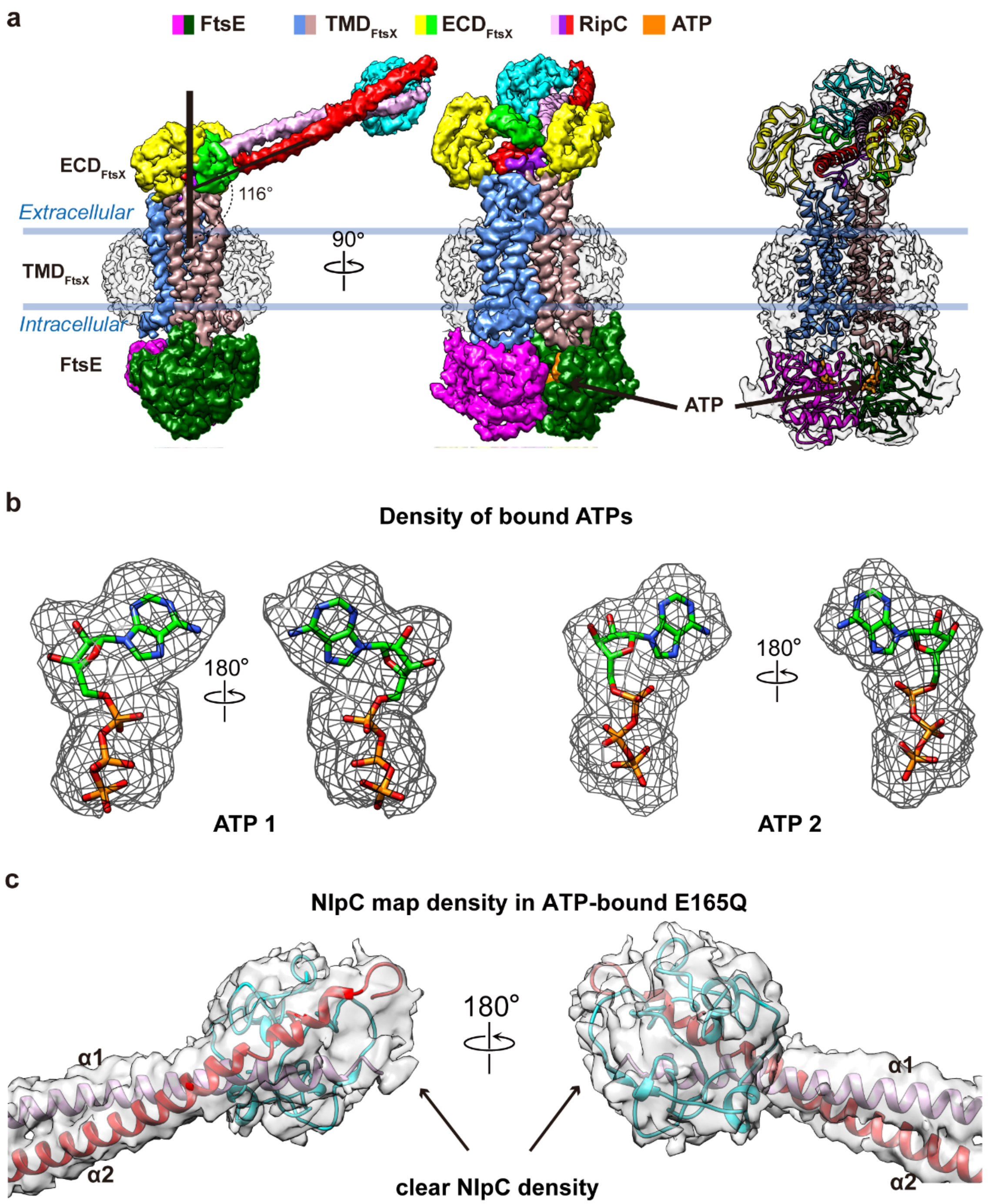

Supplementary Figure 10. Cryo-EM density maps and model fitting of the FtsE^{E165Q}X-RipC complex in the presence of ATP. a, Side- and front- views of the cryo-EM density maps of FtsE^{E165Q}X-RipC in the presence of ATP (left and middle), along with the ribbon representation of the structure model (right). The color scheme is as follows: FtsX in cornflower blue/rosy brown, FtsE in magenta/dark green, ATP in orange, the α1 helix of bound RipC in plum, the α2 helix of bound RipC in red, and the lip region in between colored in purple, the NlpC catalytic domain in cyan. **b,** The cryo-EM density map of bound-nucleotide in the FtsE^{E165Q}X-RipC complex. The conformation is identified as ATP and exhibits a good fit. **c** Clear density of the NlpC domain is

observed in $FtsE^{E165Q}$X-RipC complex map. The EM density of ATP-bound $FtsE^{E165Q}$X-RipC is in gray with 60% transparency.

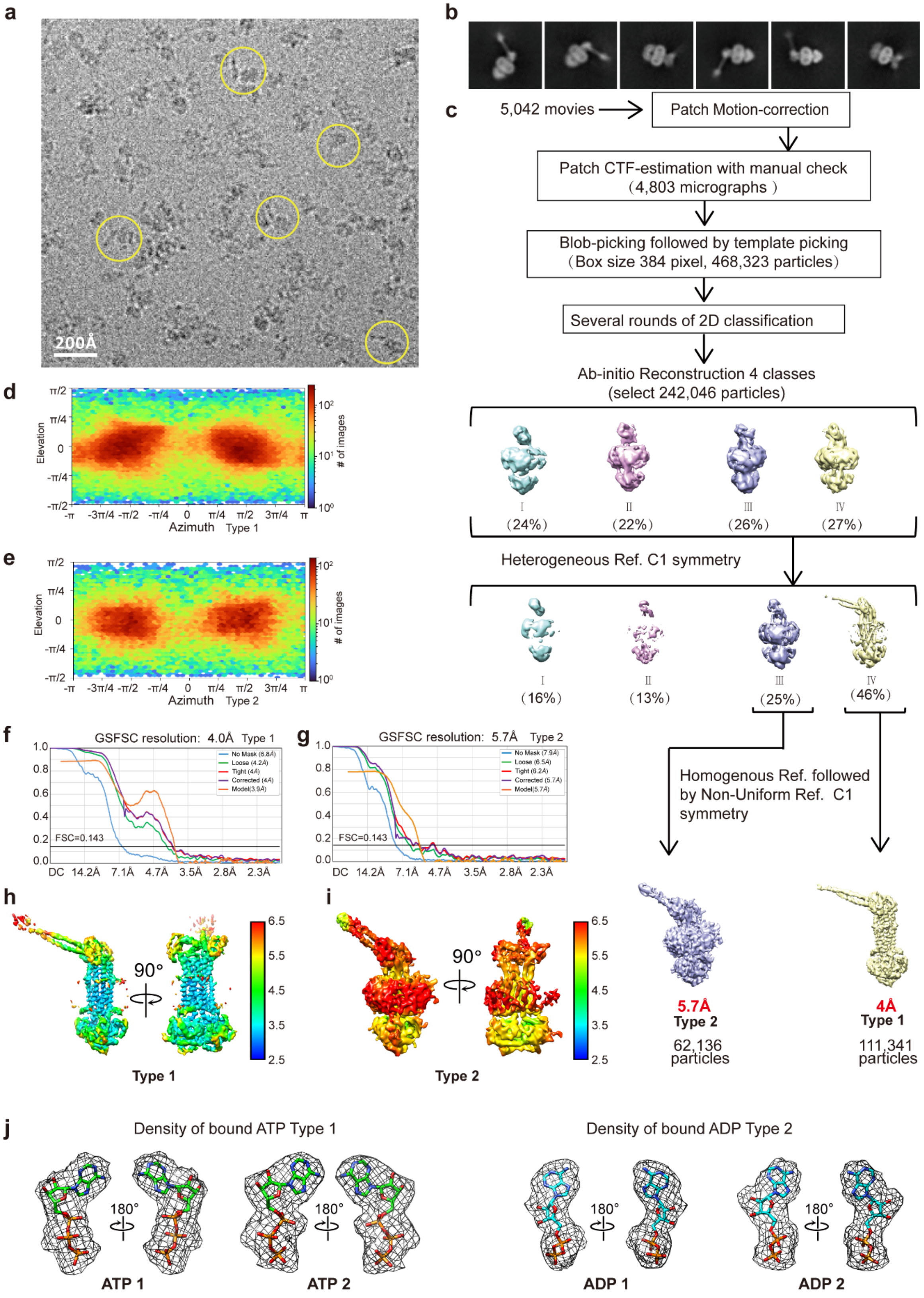

Supplementary Figure 11. Single-particle cryo-EM analysis of FtsEX-RipC complex in the presence of ATP with two major conformations elucidated. a, Representative cryo-EM image

with several particles marked by yellow circles. **b,** 2D averages of cryo-EM particle images. The box dimension is 400 Å. **c,** Image processing flowchart. The final maps of two major conformations with their overall resolutions are indicated in red. **d** and **e,** Angular distribution of the cryo-EM particles included in the final 3D reconstruction for the two major conformations. **f** and **g,** The Fourier shell correlation (FSC) curve: gold standard FSC between two half data maps with indicated resolution at FSC=0.143 (FSC corrected applied). Source data are provided as a Source Data file. **h and i,** The surface cryo-EM map was filtered to the estimated overall resolution and colored according to local resolution for the two types of conformation. **j,** The cryo-EM density map of bound-nucleotide in the two major conformational types of the FtsEX/RipC complex. The Type 1 conformation (left) is identified as ATP, while the Type 2 conformation (right) is modeled with ADP and exhibits a good fit.

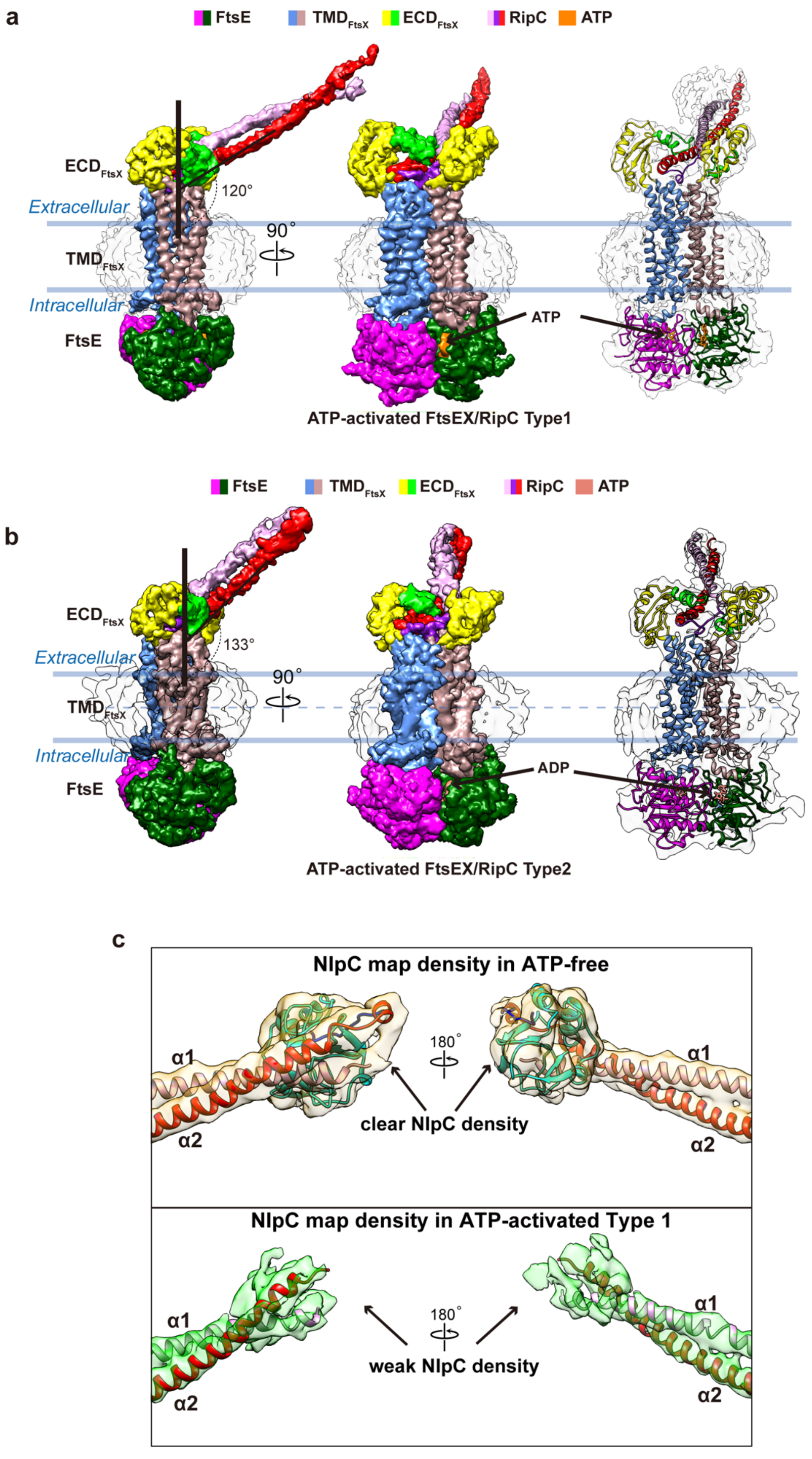
a
FtsE
TMDFtsX
ECDFtsX
RipC
ATP
ECDFtsX
120°
Extracellular
90°
TMDFtsX
Intracellular
FtsE
ATP
ATP-activated FtsEX/RipC Type1
FtsE
TMDFtsX
ECDFtsX
RipC
ATP
b
ECDFtsX
133°
Extracellular
90°
TMDFtsX
Intracellular
FtsE
ADP
ATP-activated FtsEX/RipC Type2
c
NlpC map density in ATP-free
α1
180°
α1
clear NlpC density
α2
α2
NlpC map density in ATP-activated Type 1
α1
180°
α1
weak NlpC density
α2
α2

Supplementary Figure 12. Cryo-EM density maps and model fitting of the two types of FtsEX-RipC complex in the presence of ATP. a and **b,** Side- and front- views of the cryo-EM density maps of FtsEX-RipC in the presence of ATP or ADP (left and middle), along with the ribbon representation of the structure model (right). The panel **a** is for Type 1 and the panel **b** is for Type 2. The color scheme is as follows: FtsX in cornflower blue/rosy brown, FtsE in magenta/dark green, ATP in orange, ADP in salmon, the α1 helix of bound RipC in plum, the α2 helix of bound RipC in red, and the lip region in between colored in purple. **c,** The difference of NlpC/P60 domain density in ATP-free state and ATP-activated Type 1 state. Clear EM density of the NlpC domain is observed in the ATP-free state. A weaker density is observed in the ATP-activated Type 1 state, indicating the start of the release of the NlpC/P60 domain for activation. The EM density of the ATP-free state is in yellow with 60% transparency, ATP-activated Type 1 is shown in green.

Supplementary Table 1. Oligonucleotides used in this study

Primer	Sequence (5'-3')	template
**For construction of His_6- FtsEX**		
EX-WT F	GGCAGCCATATGGTGATGATCACCCTGGACCA	genome of Mycobacterium tuberculosis
EX-WT R	GCCGCAAGCTTCTATCGCCGCACGTAGAGGC	
**For construction of His_6-SUMO-RipC**		
RipC-WT-F	ATTGGTGGATCCAATGTGCTGGCTGATCCGG	genome of Mycobacterium tuberculosis
RipC-WT-R	GGTGCTCGAGTCAGTAACGGCGGGCGTCGT	
**For FtsE, FtsX, and RipC mutations**		
FtsE-D164A-F	CTGCTGGCCGCCGAGCCCACCGGAAACCTCG	Vector-FtsEX-WT
FtsE-D164A-R	GGTGGGCTCGGCGGCCAGCAGTACCAGCGG	
FtsE-E165Q-F	CTGGCCGACCAGCCCACCGGAAACCTCGAC	Vector-FtsEX-WT
FtsE-E165Q-R	TCCGGTGGGCTGGTCGGCCAGCAGTACCAG	
FtsX-Y52A-F	CCGGGCCATCGCTCTCGACCGGGTGGAATC	Vector-FtsEX-WT
FtsX-Y52A-R	CCGGTCGAGAGCGATGGCCCGGGAGCTGTC	
FtsX-R55A-F	CTATCTCGACGCGGTGGAATCTCAGGTCT	Vector-FtsEX-WT
FtsX-R55A-R	AGATTCCACCGCGTCGAGATAGATGGCCCG	
FtsX-F61A-F	ATCTCAGGTCGCTCTCACCGAAGACGTTTC	Vector-FtsEX-WT
FtsX-F61A-R	TTCGGTGAGAGCGACCTGAGATTCCACCCG	
FtsX-F110A-F113A-F	GCAAGGCTCCCCAGGCCAAGGACGTGGCGGGCAAGG	Vector-FtsEX-WT
FtsX-F110A-F113A-R	TCCTTGGCCTGGGGAGCCTTGCGGATGGCATCGTCAT	
FtsX-F61A-F110A-F113A-F	ATCTCAGGTCGCTCTCACCGAAGACGTTTC	Vector-FtsX-F110A-113A
FtsX-F61A-F110A-F113A-R	TTCGGTGAGAGCGACCTGAGATTCCACCCG	
FtsX-F122A-F	CAAGGATTCGGCCCCGGCGTCGTTCATTGT	Vector-FtsEX-WT
FtsX-F122A-R	CGACGCCGGGGCCGAATCCTTGCCCGCCAC	
FtsX-R161A-F	GCTGATTGACGCGCTGTTCGCGGTCCTTGA	Vector-FtsEX-WT
FtsX-R161A-R	CGCGAACAGCGCGTCAATCAGCTCCTTTTG	
FtsX-Kink-2A-F (54-55 replaced by Ala)	GCCGCGGTGGAATCTCAGGTCTTTCTCACCGAAGA	Vector-RipC-WT
FtsX-Kink-2A-R (54-55 replaced by Ala)	TGAGATTCCACCGCGGCGAGATAGATGGCCCGGGA	
FtsX-Kink-3A-F (51-53 replaced by Ala)	GCCGCTGCCGACCGGGTGGAATCTCAGGTCT	Vector-RipC-WT
FtsX-Kink-3A-R (51-53 replaced by Ala)	ACCCGGTCGGCAGCGGCGGCCCGGGAGCTGTCGG	
FtsX-Kink-7A-F (49-55 replaced by Ala)	GCTGCAGCCGCGGCTGCCGCAGTGGAATCTCAGGTCTTTCTC	Vector-RipC-WT
FtsX-Kink-7A-R (49-55 replaced by Ala)	TGCGGCAGCCGCGGCTGCAGCGGAGCTGTCGGCCAACCGGACC	
RipC-R115A-F	CATGGGTGGTGCTACCCACGGCATGGATGC	Vector-RipC-WT
RipC-R115A-R	GCCGTGGGTAGCACCACCCATGTAGGTAGC	

RipC-D120A-F	CACGGCATGGCTGCGATCCTGACGGCGGAG	Vector-RipC-WT
RipC-D120A-R	CAGGATCGCAGCCATGCCGTGGGTACGACC	
RipC-Q129A-F	GAGTCCCCGGCACTGTTGATCGATCGGCTA	Vector-RipC-WT
RipC-Q129A-R	GATCAACAGTGCCGGGGACTCCGCCGTCAG	
RipC-R134A-F	GTTGATCGATGCGCTATCGGTACAGCGGGT	Vector-RipC-WT
RipC-R134A-R	TACCGATAGCGCATCGATCAACAGTTGCGG	
RipC-Delta-N59-F	TTGGTGGATCCGCGCTGCACAGTGCGCAGC	Vector-RipC-WT
RipC-Delta-N59-R	TGTGCAGCGCGGATCCACCAATCTGTTCTCT	
RipC-M141A-F	ACAGCGGGTGGCGGCGCATCAAATGTCCACG	Vector-RipC-WT
RipC-M141A-R	TGATGCGCCGCCACCCGCTGTACCGATAGC	
RipC-E59A-F	AGACCACCGCGGCGCTGCACAGTGCGCAGC	Vector-RipC-WT
RipC-E59A-R	TGCAGCGCCGCGGTGGTCTGCTCGGCCTGCC	
RipC-Y288A-W290A-F	CCCGCCGCGGCGGGTGGTGCCGCGCCCGGCGGGT	Vector-RipC-WT
RipC-Y288A-W290A-R	CACCACCCGCCGCGGCGGGCGCGCCGACCTGCGTCAA	

Supplementary Table 2. Cryo-EM data collection, refinement and validation statistics

	FtsEX (EMDB-35362) (PDB 8IDB)	FtsEX/RipC (EMDB-35363) (PDB 8IDC)	FtsE^{E165Q}X/ RipC/ATP EMDB-36304) (PDB 8JIA)	FtsEX/RipC/ATP Type 1 EMDB-35364) (PDB 8IDD)	FtsEX/RipC/ATP Type 2 EMDB-35437) (PDB 8IGQ)
**Data collection and processing**					
Magnification	81,000	81,000	81,000	81,000	81,000
Voltage (kV)	300	300	300	300	300
Electron exposure (e–/Å^2)	45	45	38	41	41
Defocus range (μm)	-0.5 to -2	-0.5 to -2	-0.5 to -2	-0.5 to -2	-0.5 to -2
Pixel size (Å)	1.06	1.06	1.06	1.06	1.06
Symmetry imposed	C1	C1	C1	C1	C1
Initial particle images (no.)	468,703	209,919	800,159	242,046	242,046
Final particle images (no.)	111,140	83,984	256,362	11,1341	62136
Map resolution (Å)	3.9	3.9	3.9	4.0	5.7
FSC threshold	0.143	0.143	0.143	0.143	0.143
Map resolution range (Å)	3.0-6.5	2.8-6.5	3.0-6.5	2.9-6.5	4.5-7
**Refinement**					
Initial model used	Alphafold*	8IDB	8IDC	8IDC	8IDC
Model resolution (Å)					
FSC threshold	0.143	0.143	0.143	0.143	0.143
Model resolution range (Å)	3.8	3.9	3.4	3.9	5.7
Map sharpening *B* factor (Å^2)	-158	-92	-165	-91.8	-410
Model composition					
Non-hydrogen atoms	6835	9701	9587	8768	8599
Protein residues	982	1342	1327	1199	1168
Ligands	NA	NA	2ATP	2ATP	2ADP
B factors (Å^2)					
Protein	129.31	216.19	126.54	153.53	220.8
Ligand	NA	NA	155.49	160.18	34.74
R.m.s. deviations					
Bond lengths (Å)	0.005	0.004	0.006	0.004	0.004
Bond angles (°)	1.077	1.034	1.108	1.049	1.093
Validation					
MolProbity score	1.68	1.79	1.8	1.63	1.82
Clashscore	5	6	6	5	7
Poor rotamers (%)	0	0	0	0	0
Ramachandran plot					
Favored (%)	93.70	92.86	92.78	95.21	93.78
Allowed (%)	5.89	6.92	7	4.79	6.04
Disallowed (%)	0.41	0.23	0.23	0	0.17

Alphafold*: The initial models used were obtained from the Alphafold under code:
AF-A0A045JB98-F1 [https://alphafold.ebi.ac.uk/entry/A0A045JB98] (*Mtb*FtsE);
AF-A0A045GRS5-F1 [https://alphafold.ebi.ac.uk/entry/A0A045GRS5] (*Mtb*FtsX);
AF-P9WHU3-F1 [https://alphafold.ebi.ac.uk/entry/P9WHU3] (*Mtb*RipC).
