# Supplementary material for: Regulation of the cell division hydrolase RipC by the FtsEX system in Mycobacterium tuberculosis
Source: Nat Commun. 2023 Dec 4;14:7999. doi: 10.1038/s41467-023-43770-6 (PMC10694151; doi:10.1038/s41467-023-43770-6)
Supplement: Supplementary file 3 — Description of Additional Supplementary Files [file 41467_2023_43770_MOESM3_ESM.pdf]

Description of Additional Supplementary Files:

Supplementary Movie 1: 3D Variability Analysis of Type 1 FtsEX-RipC complex in the presence of ATP. Surface map showing the discrete and continuous heterogeneity of cryo-EM data sets of Type 1 FtsEX-RipC complex in the presence of ATP. Hydrolysis of ATP triggers RipC tilt and NlpC domain density becomes weak. Surface in cornflower blue, and FtsE, TMDFtsX, and ECDFtsX, use arrows to identify.

Supplementary Movie 2: 3D Variability Analysis of Type 2 FtsEX-RipC complex in the presence of ATP. Surface map showing the discrete and continuous heterogeneity of cryo-EM data sets of Type 2 FtsEX-RipC complex in the presence of ATP. RipC is further tilted and the density of NlpC domain disappears. Surface in rosy brown, and FtsE, TMDFtsX, and ECDFtsX, use arrows to identify.

Supplementary Movie 3: Mechanotransmission that leads to the activation of RipC. Cartoon model showing ATP hydrolysis triggers activation of RipC. The different domains are distinguished by color. Color scheme: FtsE in magenta/ dark green, TMDFtsX in cornflower blue/rosy brown, the upper lobe of ECDFtsX in yellow, and the lower lobe of ECDFtsX in green. ATP in orange, the α1 helix of bound RipC in plum, the α2 helix of bound RipC in red, and the lip region in between colored in purple, the NIpC catalytic domain in cyan.

Supplementary Movie 4: RipC recognition through rearrangement of the ECD domain. Cartoon model showing RipC recognition through rearrangement of the one side ECD domain. The different domains are distinguished by color. Color scheme: FtsE in magenta/ dark green, TMDFtsX in cornflower blue/rosy brown, the upper lobe of ECDFtsX in yellow, and the lower lobe of ECDFtsX in green. The α1 helix of bound RipC in plum, the α2 helix of bound RipC in red, and the lip region in between colored in purple, the NIpC catalytic domain in cyan.
